# Supplementary material for: A GPCR screening in human keratinocytes identifies that the metabolite receptor HCAR3 controls epithelial proliferation, migration, and cellular respiration
Source: bioRxiv. 2023 May 31:2023.05.30.542853. Preprint. [Version 1] doi: 10.1101/2023.05.30.542853 (PMC10312554; doi:10.1101/2023.05.30.542853)

bioRxiv preprint doi: https://doi.org/10.1101/2023.05.30.542853; this version posted May 31, 2023. The copyright holder for this preprint (which was not certified by peer review) is the author/funder. This article is a US Government work. It is not subject to copyright under 17 USC 105 and is also made available for use under a CC0 license.

Supplementary Figure Legends:

Figure S1: **(A)** Quantification of EdU-proliferation assay performed 72h after siRNA transfection with the indicated GPCRs siRNAs (Class A left panel, non-Class A right panel) in differentiated primary HEK cells (48h differentiation). Results are normalized and compared to a non-targeting siRNA, represented as a black dotted line. The values of ±20% change in proliferation are shown as red dotted lines. n=4-5 independent experiments. **(B)** qRT-PCR analysis showing the expression of the selected GPCRs in HEK cells 72 h after siRNA transfection. Values are indicated as fold over non-targeting siRNA control, shown as a black dotted line. Solid color bar represents pooled siRNA, and gray bars are the results of each individual siRNAs (A-D). **(C)** Quantification of EdU-proliferation assays performed 72h after siRNA transfection with the indicated GPCRs siRNAs in primary HEK cells. Solid color bar represents pooled siRNA, and gray bars are the results of each individual siRNAs. Red indicates the assay was performed in non-differentiated conditions; blue indicates the assay was under differentiated conditions. Results are presented as in A. n=4 independent experiments. **(D)** qRT-PCR analysis showing the expression of the indicated GPCRs in N/TERT2G cells 72h after transfection with the corresponding siRNA. Values are presented as in B. **(E)** Quantification of EdU-proliferation assays in differentiated N/TERT2G cells transfected with the inidicated siRNAs. Results are presented as in A. n=5 independent experiments. **(F)** Expression of the indicated GPCRs as normalized transcripts per million (nTPM) in data from single-cell mRNA expression from human skin. Different cell populations are shown based on their corresponding cluster (indicated as "c") derived from clustering analysis. Data obtained from v22.proteinatlas.org. In A, C and E: no asterisk $p>0.05$; *$p<0.05$; **$p<0.01$; ***$p< 0.001$; ****$p< 0.0001$; 2-way ANOVA followed by t-test.

bioRxiv preprint doi: https://doi.org/10.1101/2023.05.30.542853; this version posted May 31, 2023. The copyright holder for this preprint (which was not certified by peer review) is the author/funder. This article is a US Government work. It is not subject to copyright under 17 USC 105 and is also made available for use under a CC0 license.

Figure S2: **(A)** Principal component analysis (PCA) showing the results for the RNAseq data in HEK cells transfected with the indicated siRNAs. (n=6 independent samples for siCTRL and n=3 independent samples for each siGPCR). **(B)** Pearson correlation coefficient and clustering among individual samples from the RNAseq experiment. r indicates repetition (independent sample). **(C)** Graph showing the fold change (Log2 FC) of genes expressed in the different layers in the epidermis for each of the siGPCRs datasets. Groups I-VIII contain genes clustered according to their expression pattern during differentiation, with I representing the basal compartment and VIII representing the most differentiated layer. Examples of representative genes are shown to the left. **(D)** Western blot showing keratin 10 (K10) and KLF4 expression in primary HEK or N/TERT2G cells 72h after transfection with the indicated siRNAs.

Figure S3: **(A)** Images showing expression of inducible (i) GPCRs in N/TERT2G treated (+Dox) or not (-Dox) with doxycycline for 24 h. GPCRs were tagged with N-terminal HA. HA signal is shown in red, and nuclei are shown in blue. Scale bar: 100μm. **(b)** Western blot showing the levels of Keratin 10 (K10) in N/TERT2G in cells with GPCRs-inducible expression treated (+Dox) or not (-Dox) with doxycycline for 96 h under differentiation conditions.

Figure S4: **(A)**, **(B),** and **(C)** Quantification of scratch assays in primary HEK (top) and N/TERT2G cells (bottom) for the indicated GPCRs knockdowns. The scratch area was quantified at the indicated times and normalized to t=0. n= 4-5 independent experiments performed in triplicates. **(D)** Western blot showing the levels of E-cadherin in HEK and N/TERT2G cells 72h after transfection with siRNAs.

Figure S5: **(A)** qRT-PCR analysis of the indicated genes in N/TERT2G cells 72h after siRNA transfection. Values are displayed as fold over non-targeting siRNA, shown as a black dotted line. **(B)** Quantification of EdU-proliferation assay performed 7 h after siRNA transfection with the indicated siRNAs in N/TERT2G cells. Results are normalized and compared to a non-targeting siRNA, represented as a black dotted line. ±20% change in proliferation is shown as red dotted lines. n=4 independent experiments performed in triplicates. **(C)** Quantification of scratch assays in N/TERT2G cells transfected with the indicated siRNAs for 72h. The scratch area was quantified at the indicated times and normalized to t=0. n= 3-6 independent experiments performed in triplicates. Red dotted lines indicate the values of siCTRL (non-targeting siRNA) at the different time points. **(D)** Quantification of the area under the curve (AUC) from E. **(E)** qRT-PCR analysis showing the expression of indicated genes in N/TERT2G cells 72h after siRNA transfection. Values are displayed as fold-over siCTRL (non-targeting control) transfected cells, shown as a black dotted line. **(F)** Quantification of EdU-proliferation assay performed 72h after siRNA transfection with the indicated siRNAs in N/TERT2G cells presented as in B. n= 4 independent experiments performed in triplicates. **(G)** Quantification of scratch assays in N/TERT2G cells transfected

bioRxiv preprint doi: https://doi.org/10.1101/2023.05.30.542853; this version posted May 31, 2023. The copyright holder for this preprint (which was not certified by peer review) is the author/funder. This article is a US Government work. It is not subject to copyright under 17 USC 105 and is also made available for use under a CC0 license.

with the indicated siRNAs for 72h presented as in C. n= 2-5 independent experiments performed in triplicates. **(H)** Quantification of the area under the curve (AUC) shown on (G). B, D, F, and H: no asterisk $p>0.05$; *$p<0.05$; ****$p< 0.0001$; B and F: one-way ANOVA followed by t-test; D and H: one-way ANOVA followed by t-test.

Supplementary Material Legends:

Movie S1: Representative movie of scratch assay for cells treated with siHCAR3 or non-targeting siRNA (siCtrl). Each frame was acquired every 15 min, movie is 24 hs. Scale bar: 100 µm

Movie S2: Representative movie of scratch assay for inducible HCAR3 cells treated or not with doxycycline (DOX) to induce expression of the receptor. Each frame was acquired every 15 min, movie is 24 hs. Scale bar: 100 µm

bioRxiv preprint doi: https://doi.org/10.1101/2023.05.30.542853; this version posted May 31, 2023. The copyright holder for this preprint (which was not certified by peer review) is the author/funder. This article is a US Government work. It is not subject to copyright under 17 USC 105 and is also made available for use under a CC0 license.

Fig S1

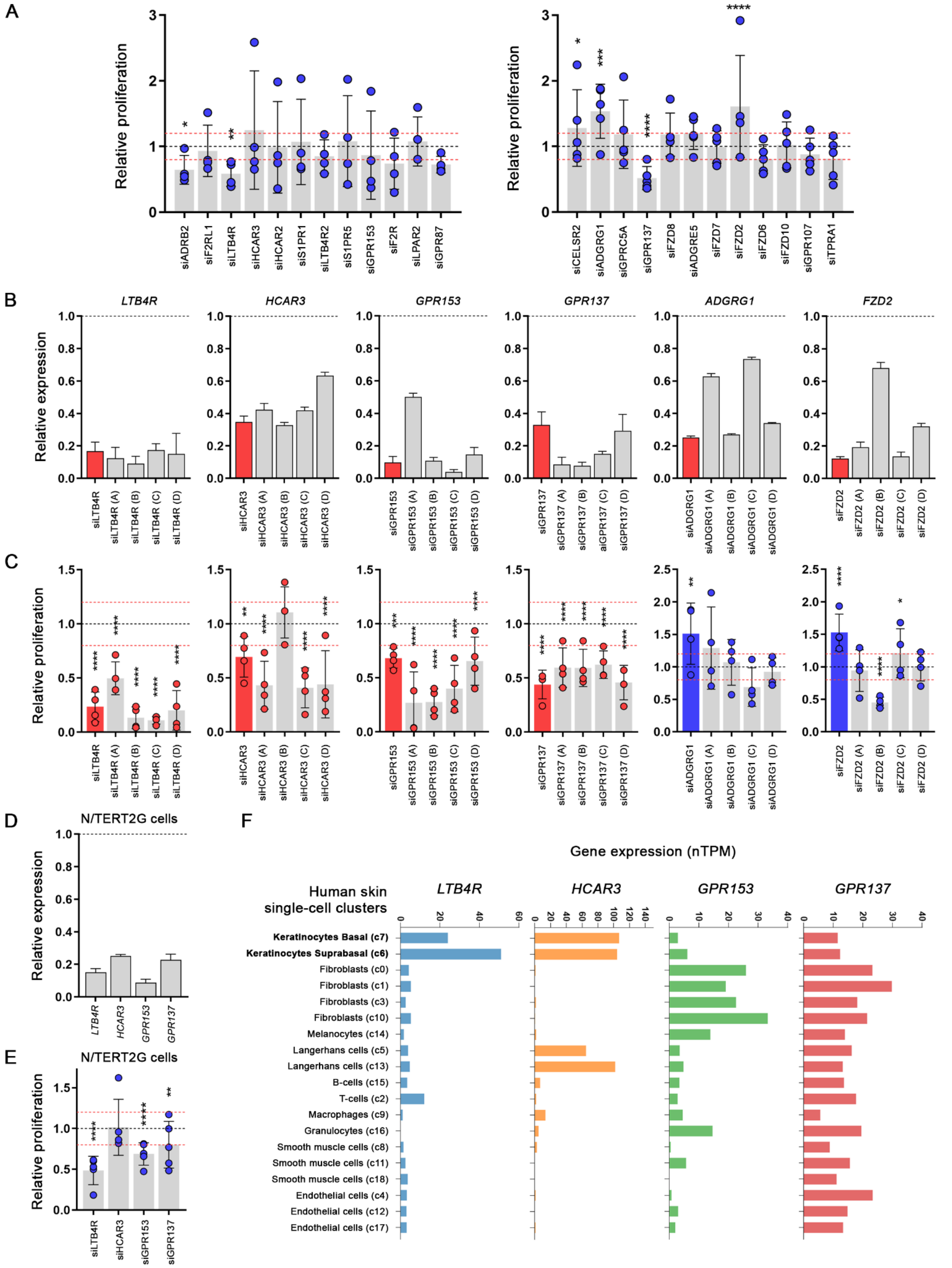

bioRxiv preprint doi: https://doi.org/10.1101/2023.05.30.542853; this version posted May 31, 2023. The copyright holder for this preprint (which was not certified by peer review) is the author/funder. This article is a US Government work. It is not subject to copyright under 17 USC 105 and is also made available for use under a CC0 license.

Fig S2

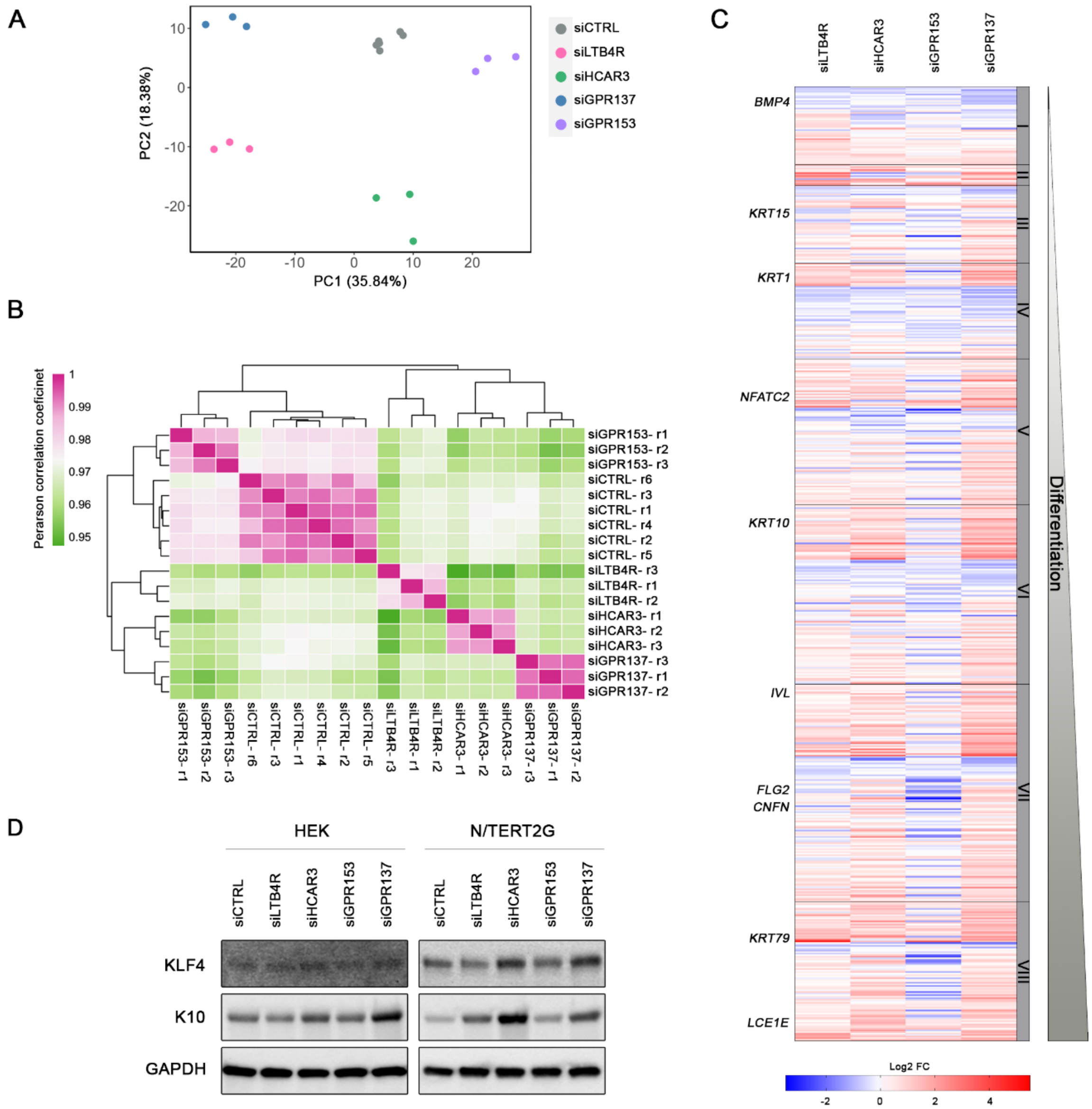

bioRxiv preprint doi: https://doi.org/10.1101/2023.05.30.542853; this version posted May 31, 2023. The copyright holder for this preprint (which was not certified by peer review) is the author/funder. This article is a US Government work. It is not subject to copyright under 17 USC 105 and is also made available for use under a CC0 license.

Fig S3

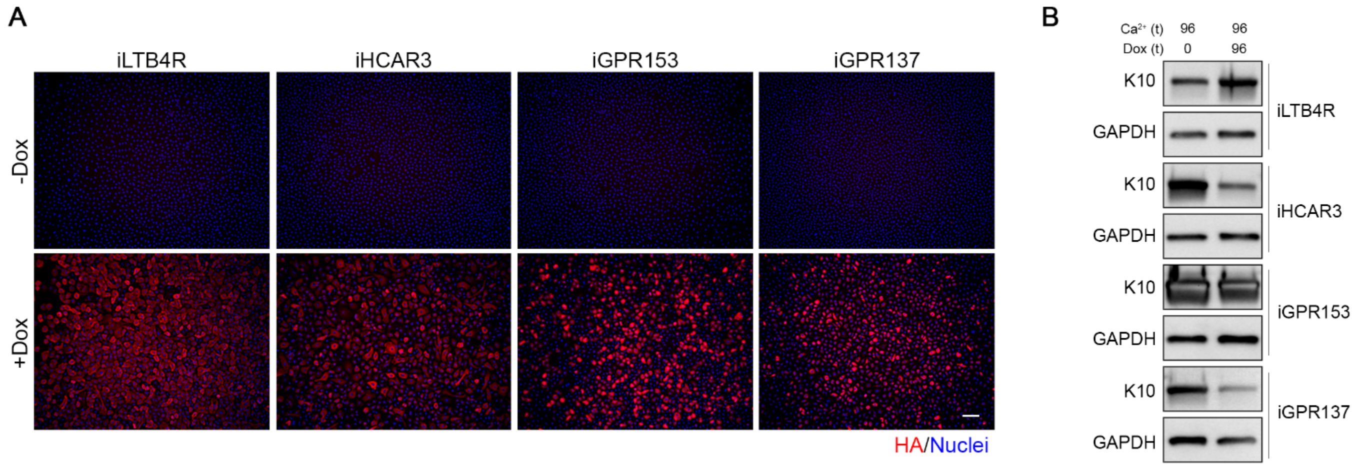

bioRxiv preprint doi: https://doi.org/10.1101/2023.05.30.542853; this version posted May 31, 2023. The copyright holder for this preprint (which was not certified by peer review) is the author/funder. This article is a US Government work. It is not subject to copyright under 17 USC 105 and is also made available for use under a CC0 license.

Fig S4

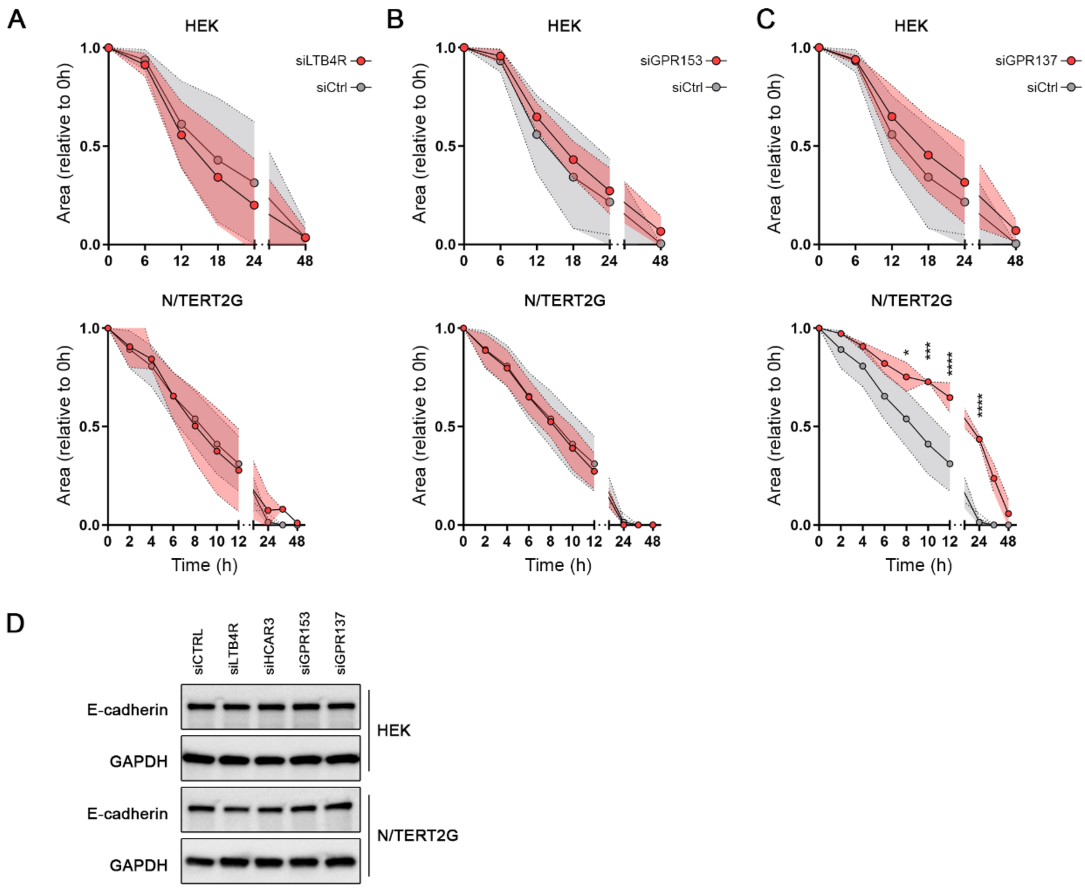

bioRxiv preprint doi: https://doi.org/10.1101/2023.05.30.542853; this version posted May 31, 2023. The copyright holder for this preprint (which was not certified by peer review) is the author/funder. This article is a US Government work. It is not subject to copyright under 17 USC 105 and is also made available for use under a CC0 license.

Fig S5

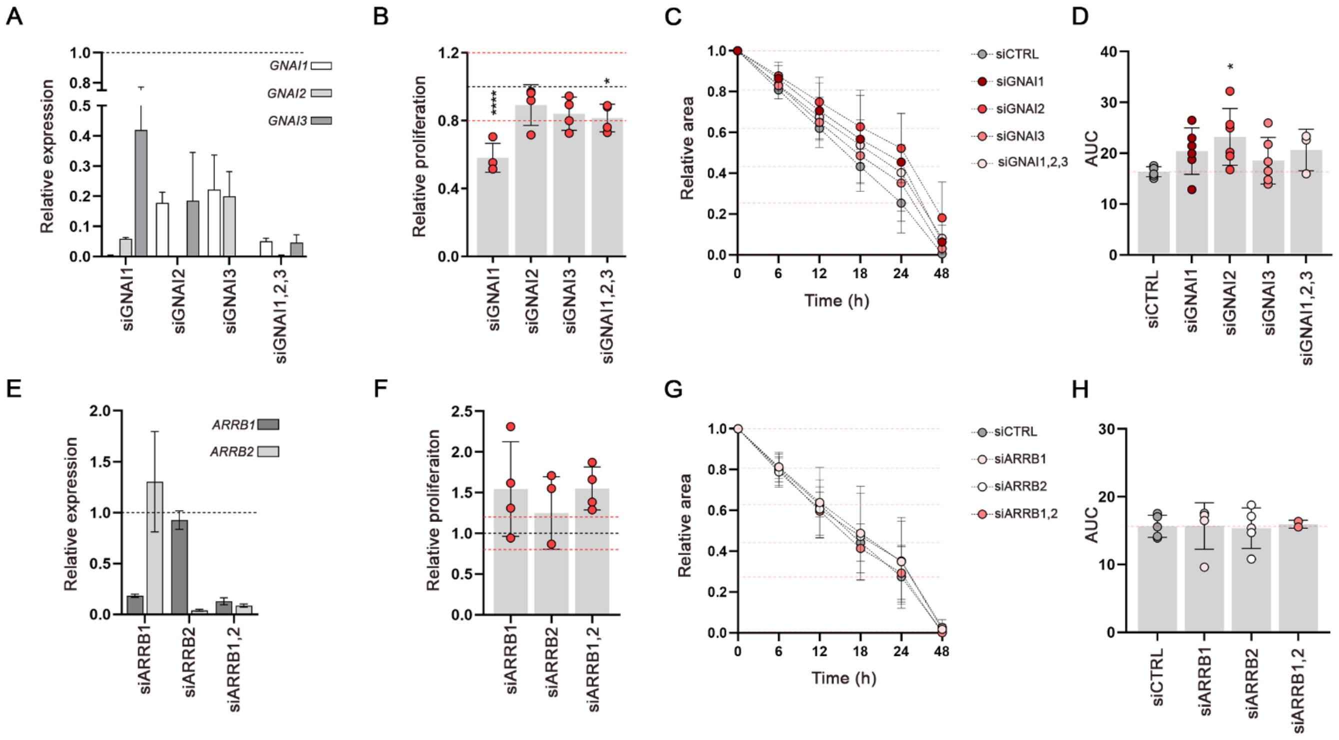

Supplement: 1 [file NIHPP2023.05.30.542853V1-supplement-1.pdf]
